# Monounsaturated fatty acids promote cancer radioresistance by inhibiting ferroptosis through ACSL3

**DOI:** 10.1038/s41419-025-07516-0

**Published:** 2025-03-18

**Authors:** Yulin Cao, Jiuming Li, Ying Chen, Yuanben Wang, Zhiang Liu, Liuying Huang, Bingxin Liu, Yuyang Feng, Surui Yao, Leyuan Zhou, Yuan Yin, Zhaohui Huang

**Affiliations:** 1 Wuxi Cancer Institute, Affiliated Hospital of Jiangnan University, Wuxi, Jiangsu 214062 China; 2 Laboratory of Cancer Epigenetics, Wuxi School of Medicine, Jiangnan University, Wuxi, Jiangsu China; 3 Department of Radiation Oncology, Affiliated Hospital of Jiangnan University, Wuxi, Jiangsu China

**Keywords:** Cancer therapeutic resistance, Radiotherapy, Rectal cancer, Oncogenes

## Abstract

Radioresistance is a major challenge in tumor radiotherapy and involves in a mixture of cellular events, including ferroptosis, a new type of programmed cell death characterized by the excess accumulation of iron-dependent lipid peroxides. In the present study, we observed that surviving cancer tissues and cells after radiotherapy had significantly greater glutathione to oxidized glutathione (GSH/GSSG) ratios and lower lipid reactive oxygen species (ROS) and malondialdehyde (MDA) levels than nonirradiated tumors and cells. Untargeted lipidomic analyses revealed that oleic acid (OA) and palmitoleic acid (POA) were the most significantly upregulated unsaturated fatty acids in irradiated surviving cancer cells compared with those in control cancer cells irradiated with IR. Both OA and POA could protect cancer cells from the killing effects of the ferroptosis inducer erastin and RSL3, and OA had a stronger protective effect than POA, resulting in lower lipid ROS production than POA. Mechanistically, OA protected cells from ferroptosis caused by the accumulation of polyunsaturated fatty acid-containing phospholipids in an ACSL3-dependent manner. A mouse model demonstrated that ACSL3 knockdown combined with imidazole ketone erastin synergistically enhanced antitumor effects in radiation-resistant tumors in vivo. Our study reveals previously undiscovered associations between radiation and fatty acid metabolism and ferroptosis, providing a novel treatment strategy for overcoming cancer radioresistance.

## Introduction

Radiotherapy (RT) has been clinically applied to treat more than 50% of solid cancers, either alone or in combination with other anticancer therapies. Despite technological advances in RT, including a more precise delivery of radiation while progressively minimizing the impact on normal tissues, radioresistance remains to be a main obstacle to improving RT efficacy. Thus, to overcome radioresistance, the interaction between radiation and abnormal biological changes in tumor cells that induce radioresistance urgently needs to be elucidated.

The mechanisms mediating radioresistance often involve the activation of DNA repair pathways and the inhibition of apoptosis [1–3]. In addition to causing DNA damage, RT also generates reactive oxygen species (ROS), which can induce oxidative damage to cell components including lipid membranes [4, 5]. Ionizing radiation (IR) can induce lipid peroxidation [6], then excessive lipid peroxidation leads to ferroptosis [7]. In recent years, the contribution of ferroptosis to radiosensitization has attracted increased amounts of attention [3, 8, 9]. Several studies have confirmed that ferroptosis is an important factor mediating the RT-induced cell death response, and that ferroptosis resistance contributes to radioresistance and the failure of RT [1, 2, 10, 11]. However, the interaction between radioresistance and radiation-mediated ferroptosis has not yet been fully elucidated.

Ferroptosis, a new type of programmed cell death, is triggered by oxidative stress and characterized by the accumulation of iron-dependent lipid peroxides to a lethal level [3, 12–14]. Ferroptosis has been identified as a crucial mechanism involved in tumor progression [15, 16]. Iron-catalyzed hyperoxidation of phospholipids containing polyunsaturated fatty acids (PUFA-PLs) is the core feature of ferroptosis. The content of intracellular polyunsaturated fatty acids (PUFAs) determines the degree of lipid peroxidation and the susceptibility of cells to ferroptosis [9]. In contrast, monounsaturated fatty acids (MUFAs) are less susceptible to peroxidation due to the absence of the bis-allylic, and can inhibit lipid peroxidation and ferroptosis by replacing PUFAs in the cell membrane phospholipids (PLs) [17]. Therefore, cancer cells can be theoretically sensitized to ferroptosis by modulating the activities of enzymes involved in MUFA-PL synthesis, such as stearoyl-CoA desaturase 1 and long-chain acyl-coenzyme A synthetase-3 (ACSL3).

Several studies have shown that the interplay between fatty acid (FA) metabolism and ferroptosis is linked to tumor aggressiveness and rapid progression [18–20]. Moreover, IR can regulate both ferroptosis and FA metabolism [21]. However, the specific mechanism linking IR-induced ferroptosis with FA metabolism is still unknown. Interestingly, IR can influence several key processes of FA metabolism, including synthesis, transport, oxidation, and reduction. The unsaturated FA concentration was reported to increase significantly following irradiation in glioblastoma [22]. Recent studies have revealed that the FA saturation level of PLs is an important determinant of ferroptosis sensitivity, with MUFA-PLs promoting ferroptosis resistance and PUFA-PLs promoting ferroptosis sensitivity [23, 24]. Therefore, the effects of FA metabolism, which is implicated in ferroptosis, on radiosensitization require additional attention.

In this study, we showed that irradiated surviving cancer cells undergo less ferroptosis than their unirradiated control cells and exhibit increased radioresistance in human cancers. These effects were mediated mainly by OA in an ACSL3-dependent manner via the inhibition of radiation-induced ferroptosis. Notably, we also found that ACSL3 knockdown and imidazole ketone erastin (IKE, an inducer of ferroptosis) synergistically restored the sensitivity of radioresistant cancer cells to radiation both in vitro and in vivo. Our work therefore provides a new therapeutic target and strategy for overcoming radioresistance in cancer patients.

## Materials and methods

### Cell culture and irradiated surviving progeny cell models

SW837, CMT93, A549, and H1299 cells were obtained from the American Type Culture Collection (ATCC). These cell lines were authenticated in this study. All cell lines were confirmed as Mycoplasma negative before experiments. These cells were maintained in high-glucose DMEM containing 10% fetal bovine serum (FBS) and penicillin/streptomycin at 37°C in a humidified atmosphere containing 5% CO_2_. SW837, CMT93, A549, and H1299 cells were cultured to 80% confluence and then subjected to X-ray irradiation (12, 22, 15, or 15 Gy, respectively) at a dose rate of 3 Gy/min using a linear accelerator (Oncor, Siemens, Berlin, Germany). When the cells were irradiated, a T25 flask was placed on the couch, and a 1.5-cm-thick bolus was used to correct the distribution of radiation. The irradiation characteristics included the following: beam energy, 6-MV photons; source-surface distance, 100 cm; the size of the radiation field, 10 × 10 cm^2^; gantry, 180°. Dosimetry was performed with a cylindrical ionization chamber before irradiation. Radioresistant SW837, CMT93, A549, and H1299 cells (SW837-RR, CMT93-RR, A549-RR, and H1299-RR) were generated by the clonogenic selection method. Briefly, these cells were seeded at a density of 1000 cells per well (six-well plates), exposed to X-ray irradiation (12, 22, 15, or 15 Gy, respectively), and cultured for 10-14 days.

### Clinical samples

Human primary rectal cancer tissues from patients receiving neoadjuvant RT (before and after RT) were collected from Affiliated Hospital of Jiangnan University with informed consent. The study was approved by the Clinical Research Ethics Committees of Affiliated Hospital of Jiangnan University [LS2021104].

### Cell viability assay

For cell viability assays, 1000 cells per well were seeded in 96-well plates and incubated for 24 h. The cells were then treated with shACSL3-AAV (450,000 genome copies/cell), 50 µM IKE (Yuanye Biotechnology Co., Ltd, Shanghai, China), 20 nM OA (Cayman Chemical, Michigan, USA), 20 nM palmitoleic acid (POA, Cayman Chemical), 20 μM T863 (MCE), or 10 μM PF-06424439 (MCE) for 48 h and treated with 1 μM erastin (MCE) or 0.5 μM RSL3 (MCE) for 24 h. Then, the medium was replaced with 100 μL of fresh medium containing 10 μL of Cell Counting Kit-8 (CCK8) reagent (APEXBIO, Texas, USA). After the cells were incubated for 2 h in a humidified incubator (at 37 °C and 5% CO2), the absorbance was detected at a wavelength of 450 nm using a microplate reader (BioTek, Vermont, USA). The cell viability of the samples was calculated according to the manufacturer’s instructions.

### Clonogenic survival assay

Approximately 500-1000 cancer cells were seeded into each well of six-well plates and incubated for 16 h followed by treatment with different doses of IR using a linear accelerator. After approximately 14 days, the cells were washed with precooled PBS, fixed in 4% paraformaldehyde, and stained with 0.1% crystal violet. The numbers of cell clones were counted.

### Measurement of GSH/GSSG

The GSH/GSSG levels in cancer cells and tissues were measured using GSH/GSSG assay kit (Beyotime, Shanghai, China), the treated cancer cells and tissues were lysed by repeated cycle of freezing and thawing, then centrifuged to collect the supernatant for the measurement of GSH and GSSG according to the manufacturer’s protocol.

### Measurement of MDA

The MDA content was measured using MDA assay kit (Beyotime) following the manufacturer’s instructions. In brief, cancer cells and tissues lysates were centrifuged to harvest the supernatant. After MDA solution was added to each sample, the mixture was heated at 100 °C for 15 min and then centrifuged to harvest the supernatant. The OD value of each well was measured with a microplate reader at 532 nm.

### Lipid ROS assay

The level of Lipid ROS was detected by BODIPY 581/591 C11 (Thermo Fisher, Massachusetts, USA). After cells were exposed to the different treatments, 5 μM of BODIPY 581/591 C11 dye was added and incubated at 37 °C for 30 min in a dark environment following the manufacturer’s instructions. All results were analyzed using FlowJo V10 software.

### Pathway enrichment analyses

Kyoto Encyclopedia of Genes and Genomes (KEGG) enrichment analyses of the coregulated differentially expressed genes were performed through the online tool Database for Annotation, Visualization, and Integrated Discovery (DAVID) (version 6.8, https://david.ncifcrf.gov/). Differentially abundant metabolites were subjected to pathway enrichment analyses via Human Metabolome Database (HMDB), and KEGG.

### Transmission electron microscopy (TEM) analyses

Cells cultured in 6-well plates were fixed with a solution containing 2.5% glutaraldehyde in 0.1 M cacodylate buffer (pH 7.3). After washing with 0.1 M sodium cacodylate buffer, the cells were postfixed with 1% buffered osmium. After dehydration and embedding, the samples were incubated at 70 °C for 24 h. Finally, ultrathin sections were prepared and examined with a Hitachi-HT7800 TME (Japan).

### Quantitative RT-PCR (qRT-PCR)

Total RNA was reverse transcribed into cDNA using the HiFiScript cDNA Synthesis Kit (CWBIO, Beijing, China). Gene expression levels were measured by qRT-PCR using Ultra SYBR Mixture (Vazyme, Nanjing, China). Related primer sequences are listed in Supplementary Table 1.

### Western blotting

Cells were lysed using RIPA buffer (Beyotime) containing a protease inhibitor cocktail (MCE). The resulting proteins were then separated via 10% SDS-PAGE and transferred onto a PVDF membrane (Millipore, Massachusetts, USA). After blocking with 5% skim milk powder, the membranes were incubated with primary antibodies targeting ACSL3 (1:1000, Abmart, Shanghai, China), Glutathione Peroxidase 4 (GPX4, 1:1000, Proteintech, Chicago, USA), the light chain subunit solute carrier family 7 member 11 (SLC7A11, 1:1000, Proteintech), nuclear factor erythroid 2-related factor 2 (Nrf2, 1:1000, Santa Cruz, California, USA), transferrin receptor 1 (TfR1, 1:5000, Proteintech), and β-actin (1:5000, CWBIO) at 4 °C overnight, and with peroxidase-conjugated anti-rabbit secondary antibody (Jackson, Pennsylvania, USA) for 2 h. The intensity of the protein bands was visualized by an enhanced chemiluminescence assay (Vazyme) and measured using a ChampChemi-610 imaging system (SINSAGE, Beijing, China).

### CRISPR-Cas9 assay

CRISPR-Cas9-mediated knockout was performed as we previously described [25]. Briefly, guide RNAs targeting ACSL3 (sgACSL3#1 and #2) were obtained from the CRISPR library and cloned into the lentiCRISPRv2 vector. Lentiviruses were then generated by cotransfection of the lentiCRISPRv2 vector and packaging plasmids into HEK-293T cells. For gene knockout, 1 × 10^5^ cells were infected with CRISPR-Cas9-gRNA lentivirus and selected with puromycin for 7 days. Puromycin-resistant clones were isolated and confirmed by western blotting.

### Generation of SW837 and CMT93 cells with stable ACSL3 overexpression

The coding sequence of ACSL3 was inserted into pLenti-EF1a-EGFP-F2A-Puro-CMV. The obtained pLenti-EF1a-EGFP-F2A-Puro-ACSL3 plasmid was cotransfected into HEK-293T cells along with the packaging plasmid ps-PAX2 and the envelope plasmid pMD2G using Lipofectamine 2000 (Thermo Fisher) as we previously described [25]. The virus particles were harvested 48 h after cotransfection and then individually used to infect SW837 and CMT93 cells to generate corresponding stable cell lines, which were subsequently selected with puromycin for 7 days. Puromycin-resistant clones were isolated and confirmed by western blotting.

### Immunohistochemistry (IHC) staining

IHC staining was performed on 4-μm sections of paraffin-embedded tumor tissue samples to measure ACSL3 protein expression. Briefly, the slides were incubated with anti-ACSL3 antibodies (1:200, Abmart), and anti-4-hydroxy-2-noneal antibodies (4-HNE, 1:200, Abcam, Cambridge, Britain) at 4 °C overnight. The subsequent steps were performed using the GT Vision III Detection System/Mo&Rb (GeneTech, Suzhou, China). Finally, the staining results were evaluated and scored by two pathologists.

### Liquid chromatography-mass spectrometry (LC-MS) analyses

Cells (5 × 10^6^ cells) were harvested and washed twice with PBS. The cells were frozen in liquid nitrogen and thawed 3 times and sonicated on ice. The protein samples were extracted using 2 mL of methylation mixture (MeOH: benzene: DMP (2, 2-dimethoxypropane): H_2_SO_4_ = 39:20:5:2) and 1 mL heptane. Then, the supernatants were analyzed using HPLC-MS (UPLC Acquity I-Class PLUS and UPLC Xevo G2-XS QTof; Waters, Massachusetts, USA).

### Oil Red O staining

Oil Red O (Sigma-Aldrich, Missouri, USA) was dissolved in propylene glycol by heating to 95 °C and filtered through filter paper to a final concentration of 0.5% w/v. Immediately prior to staining, the Oil Red O solution was filtered through a 0.45 μm syringe filter to remove residual particulates from the solution. Cells cultured on coverslips were fixed in 4% paraformaldehyde, rinsed in PBS, and equilibrated in 100% propylene glycol for 2 min at room temperature. The cells were stained in the Oil Red O solution for 1 h at room temperature and rinsed in 85% propylene glycol to differentiate the staining. After rinsing in PBS, the cells were counterstained with hematoxylin for 1 min, rinsed, and mounted onto glass slides using Glycerol Jelly Mounting Medium (Beyotime). Images were captured by a Leica DMi8 inverted microscope and analyzed using ImageJ.

### Tumor RT in a mouse tumor model

Four-week-old male C57BL/6 mice were purchased from the Shanghai Animal Center, Chinese Academy of Sciences, and maintained under specific pathogen-free conditions. These mice were inoculated with 5 × 10^5^ A549, CMT93, or CMT93-RR cells subcutaneously into the right hind limb. When the tumor size reached approximately 100 mm^3^, the mice were assigned randomly to different groups (*n* = 5). Before radiation treatment, each mouse was anesthetized and shielded by a lead box, so that only the irradiated tumor was exposed and a linear accelerator was then used to deliver 8 Gy (or 12 Gy) radiation at a rate of 3 Gy/min. A total of 40 mg/kg IKE was administered intraperitoneally every 2 days for a total of 6 times. The shACSL3-AAV was intratumorally injected into mice with a dose of 1.8 × 10^11^ viral particles per animal. The shACSL3-AAV was designed and constructed by PackGene Biotech (Guangzhou, China). The AAV constructs were injected once every two days for a total of 6 times. All animal experiments were performed in accordance with the relevant institutional and national guidelines and the regulations of Jiangnan University Medical Experimental Animal Care Commission (JN. No20230228c0480515).

### Statistical analyses

The results are presented as mean ± standard deviation. Statistical analyses were carried out using GraphPad Prism 9.0 (GraphPad, California, USA) and SPSS 20.0 (SPSS, Chicago, USA). The sample size was determined based on preliminary data showing the variance within and between groups. Generally, we used the standard unpaired Student’s *t*-test to compare two independent groups. Analysis of variance (ANOVA) was used to compare the differences among three or more groups, and if the ANOVA showed significant differences we used Dunnett’s or Tukey’s post hoc test to identify pairs with significant differences. *P* < 0.05 was considered to be statistically significant.

## Results

### Decreased ferroptosis contributes to the radioresistance in RR cancer cells

**Fig. 1:**
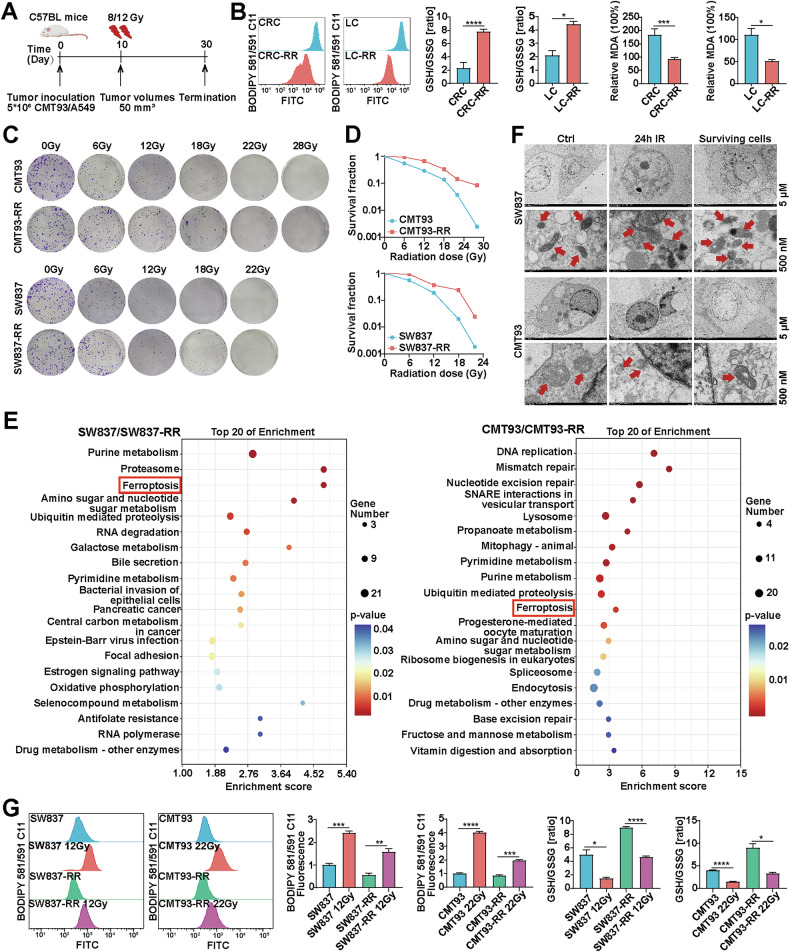
Decreased ferroptosis contributes to radioresistance in radioresistant cancer cells. **A** Treatment schedules for the CMT93 (n = 3) and A549 (*n* = 3) subcutaneous xenograft models, which were exposed to 8 Gy or 12 Gy of X-rays, respectively. **B** Quantitative analyses of lipid peroxidation and GSH/GSSG levels in nonirradiated control tumors and radioresistant tissues (RR) after RT (*n* = 3). **C**, **D** Representative images of clonogenic survival assays of CMT93 or CMT93-RR cells subjected to 0 to 28 Gy of X-ray, SW837 or SW837-RR cells subjected to 0 to 22 Gy of X-ray. The dose survival curves were plotted using GraphPad Prism 9.0 software. **E** Heatmap of differentially expressed proteins between radioresistant and control cells involved in ferroptosis. **F** TEM images of cell morphology after IR treatment (12 Gy for SW837, 22 Gy for CMT93) for 24 h; red arrows indicate mitochondria. **G** Quantitative analyses of lipid peroxidation and GSH/GSSG levels in reirradiated radioresistant cancer cells and control cells. (*n* = 3) *p < 0.05, **p < 0.01, ***p < 0.001, ****p < 0.0001.

IR-induced oxidative stress and ferroptosis are important biological effects of IR in tumor destruction, and inhibition of ferroptosis may be part of the reason for RT failure in cancer. Clinically, surviving tumor cells after IR usually exhibit increased radioresistance. To investigate the ferroptosis levels in the surviving tumor cells after RT, subcutaneous tumors of mouse rectal cancer (CMT93) and lung cancer cells (A549) from immunocompetent C57BL mice were generated and then exposed to 12 Gy or 8 Gy of X-rays, respectively (Fig. 1A). Compared with those in nonirradiated control tumors, significantly increased GSH/GSSG levels and decreased lipid ROS, MDA and Fe^2+^ levels were observed in the residual tumor tissues after RT, suggesting decreased ferroptosis levels and increased radioresistance in these tumors (Fig. 1B and Supplementary Fig. S1A). Next, we established irradiated surviving progeny cell models using two rectal cancer cell lines irradiated with sublethal doses, SW837-RR (12 Gy) and CMT93-RR (22 Gy). As shown in Fig. 1C, D, the surviving cancer cells exhibited significantly increased radioresistance. To investigate the underlying mechanisms, we compared the protein expression profiles of these radioresistant cancer cells with those of their unirradiated control cells using mass spectrometry. Differentially expressed proteins between the two groups were selected for KEGG enrichment analyses. The results showed that several pathways, especially ferroptosis, were significantly enriched (Fig. 1E). We next studied the potential role of ferroptosis in the death response of surviving tumor cells to IR. We treated multiple cancer cell lines (SW837, CMT93, A549, and H1299) and their corresponding radioresistant cells with the ferroptosis inhibitor ferrostatin-1 (5 μM), the apoptosis inhibitor Z-VAD-fmk (5 μM), the necroptosis inhibitor necrostatin-1 (2 μM), and the autophagy inhibitor 3-methyladenine (3-MA, 5 mM) for 48 h, followed by exposure to IR, and then assessed their viability. We found that the restoration of cell viability induced by ferrostatin-1 was more pronounced than that induced by other cell death inhibitors in these cancer cells. However, ferrostatin-1 did not result in greater recovery of cell viability induced by IR in radioresistant cells with their corresponding parental cells. (Figure. S1B). These results suggest that ferroptosis resistance plays a pivotal role in mediating the resistance of radioresistant cells to IR. In addition, the transmission electron microscopy (TEM) results revealed that cancer cells (SW837/CMT93) treated with IR (12/22 Gy) for 24 h exhibited shrunken mitochondria with enhanced membrane density, a morphologic feature of ferroptosis, which was obviously alleviated in their corresponding radioresistant cells after 24 h of reirradiation (Fig. 1F). What is more, the IR-induced increase in lipid ROS, Fe^2+^ levels and decrease in GSH/GSSG levels in the control cancer cells were obviously inhibited in the reirradiated radioresistant cells (Fig. 1G and Supplementary Fig. S1C). Taken together, these data suggest that ferroptosis is obviously inhibited in radioresistant cancer cells and closely associated with cancer radioresistance.

### IR alters the FA metabolism profiles of radioresistant cancer cells

**Fig. 2:**
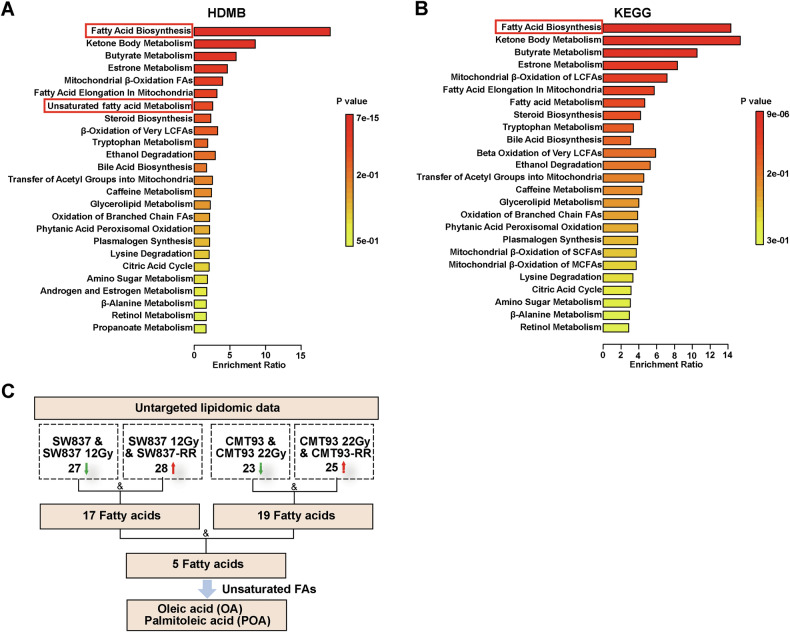
IR treatment alters the FA metabolism profiles of radioresistant cancer cells. HDMB (**A**) and KEGG (**B**) analyses show the changes in fatty acid metabolism in nonirradiated cancer cells, irradiated cancer cells, and their corresponding radioresistant cells. **C** Flow chart showing the results of the intersection of significantly changed unsaturated FAs in nonirradiated control cells, cancer cells treated with IR, SW837-RR and CMT93-RR cells.

FA synthesis and metabolism pathways are closely associated with ferroptosis [21]. To assess FA metabolism changes in irradiated cancer cells, we conducted untargeted lipidomic analyses of cancer cells (SW837/SW837-RR and CMT93/CMT93-RR) irradiated with sublethal doses and their nonirradiated control cells, and a total of 2502 lipids were identified (*n* = 3), including 91/93 downregulated lipids in irradiated SW837/CMT93 cells compared with the unirradiated control cells, and 55/66 upregulated lipids in SW837-RR/CMT93-RR cells compared with their irradiated parental cells (fold change > 2 or <0.5). All the identified lipids were annotated using the KEGG, and HMDB databases, and the top 25 annotations were selected for further analyses. Enrichment analyses revealed that the differential lipids between irradiated and nonirradiated cancer cells were enriched mainly in the pathways related to FAs, especially unsaturated FAs (Fig. 2A, B). As shown in Fig. 2C, we compared the significantly changed FAs induced by IR in different cancer cells and their corresponding radioresistant cells. In irradiated SW837 and CMT93 cells, 27 and 23 FAs were downregulated, respectively, compared to unirradiated controls. Conversely, 28 and 25 FAs were upregulated in SW837-RR and CMT93-RR cells, respectively, compared to their irradiated parental cells (fold change >2 or <0.5). By overlapping the changed FAs between these groups, we identified 5 FAs, including two MUFAs (OA and POA) (Fig. 2C). Based on these results and the pathway enrichment analyses (Fig. 2A), we focused on OA and POA for further studies. Collectively, these results indicate that IR altered the metabolism profiles of MUFAs, particularly OA and POA, in radioresistant cancer cells.

### OA protects radioresistant cells from ferroptosis

**Fig. 3:**
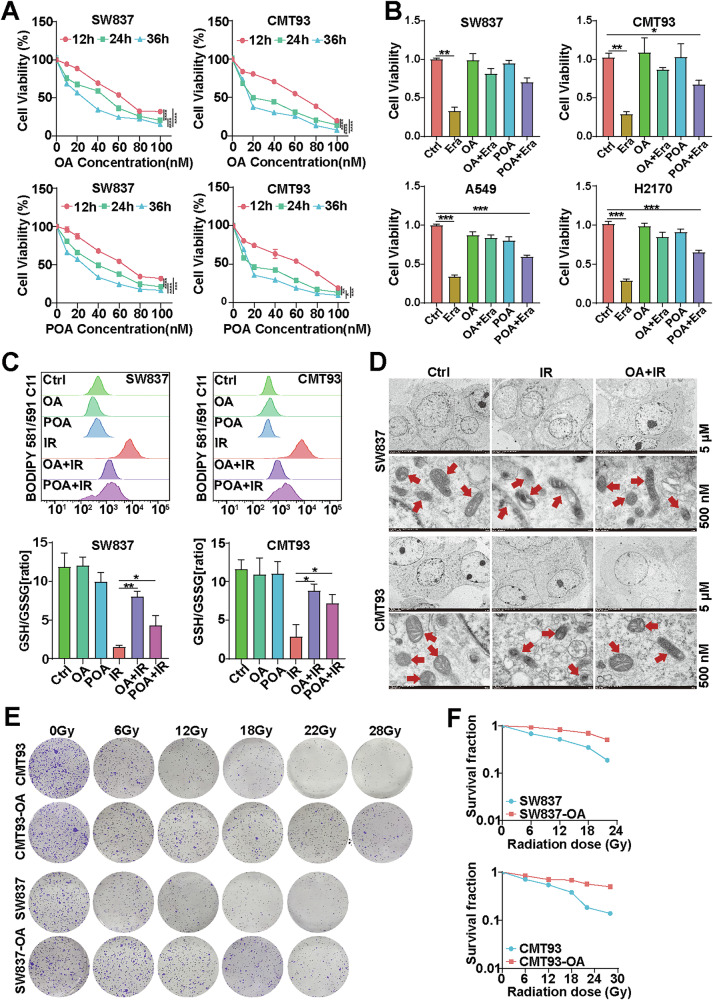
OA protects radioresistant cells from ferroptosis. **A** The cell cytotoxicity of different concentrations (0-100 nM) of OA and POA to SW837 and CMT93 cells at 12 h, 24 h, and 36 h (*n* = 3). **B** CCK-8 assays were performed to determine the effects of erastin (1 μM), OA (20 nM), and POA (20 nM) on the viability of SW837, CMT93, A549, and H1299 cells (*n* = 3). **C** Quantitative analysis of lipid peroxidation levels and the GSH/GSSG ratio in SW837, CMT93, A549, and H1299 cells treated with 1 μM erastin, 20 nM OA, or 20 nM POA (*n* = 3). **D** Cell morphology was observed via TEM after cells were treated with or without OA (20 nM) for 24 h, and then treated with IR (12 Gy for SW837, 22 Gy for CMT93) for 24 h; red arrows, mitochondria. **E** Effect of OA pretreatment on colony formation. The cells were plated in 6-well plates for 12 h and then irradiated with 0 to 28 Gy of X-ray radiation using a linear accelerator. The cells were grown at 37 °C for 14 days, after which the number of colonies containing 50 or more cells was counted. Each experiment was performed at least three times. **F** Dose survival curves of CMT93 or CMT93-RR cells subjected to 0–28 Gy of X-ray irradiation, and SW837 or SW837-RR cells subjected to 0–22 Gy of X-ray irradiation. *p < 0.05, **p < 0.01, ***p < 0.001, ****p < 0.0001.

OA and POA are two MUFAs that inhibit ferroptosis by reducing the amount/density of PUFAs available for oxidation in membranes [24, 26, 27]. To determine whether OA and POA are involved in radioresistance, we treated multiple rectal and lung cancer cell lines (SW837, CMT93, A549, and H1299) with OA (or POA) and performed an in vitro cytotoxicity assay using CCK8 (Fig. 3A and Supplementary Fig. S2A). As shown in Fig. 3B and Figure. S2B, treatment with OA or POA alone did not obviously affect cell proliferation, but both of these agents could protect cancer cells from the killing effects of erastin and RSL3. Furthermore, the cell viability of the OA+erastin (or OA + RSL3) group was greater than that of the POA+erastin (or POA + RSL3) group, indicating that OA had a stronger protective effect than POA. In addition, we revealed that OA or POA pretreatment significantly reduced lipid ROS production, Fe^2+^ levels and increased GSH/GSSG ratios, and stronger effects were observed in the OA groups compared with the POA groups in the irradiated cancer cells (Fig. 3C and Supplementary Fig. S2C). Taken together, these data suggest that both OA and POA efficiently inhibit erastin (or RSL3)-induced ferroptosis in cancer cells.

Consequently, OA was selected as a representative MUFA for subsequent experiments. TEM results revealed that IR induced typical ferroptosis-related morphological changes, as indicated by a decreased mitochondrial size and increased mitochondrial membrane density compared with those in the nonirradiated control group. However, the change in ferroptosis-related morphology in the irradiated cancer cells was obviously alleviated in the OA pretreatment group (Fig. 3D). Next, we evaluated the impact of OA pretreatment on the clonogenic survival of cancer cells irradiated with different doses. The results showed that OA pretreatment partially restored the clonal survival of cancer cells that was inhibited by IR exposure (Fig. 3E, F), suggesting that OA enhances the radioresistance of cancer cells. In summary, these results indicate that OA protects cancer cells from IR-induced ferroptosis and promotes cancer radioresistance.

### OA protects radioresistant cells from ferroptosis in an ACSL3-dependent manner

**Fig. 4:**
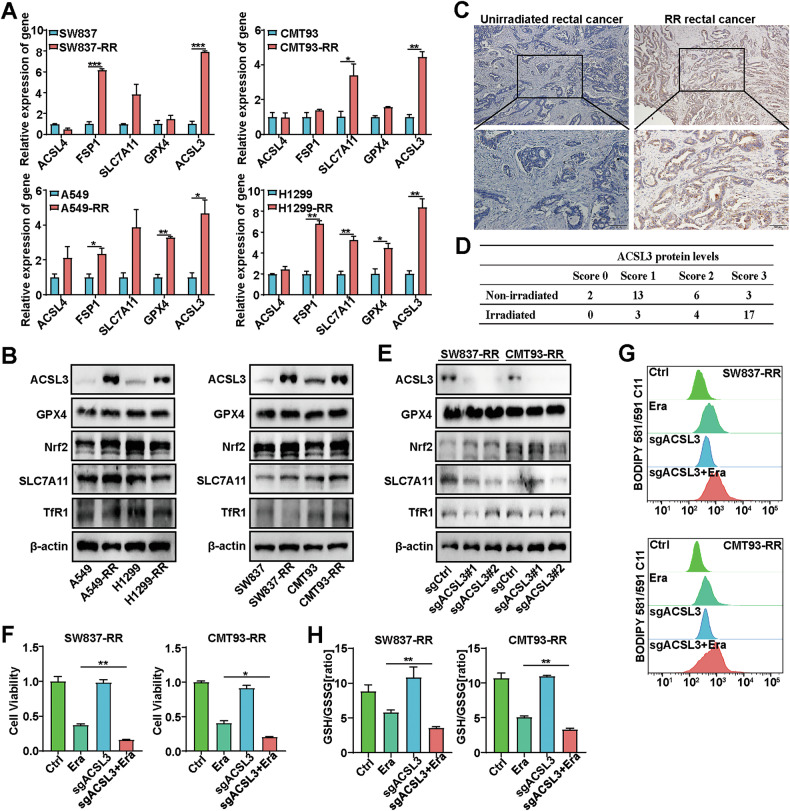
Ferroptosis resistance in radioresistant cancer cells depends on ACSL3. **A** The mRNA expression of ferroptosis-related genes in radioresistant cancer cells (SW837-RR, CMT93-RR, A549-RR, H1299-RR) and their parental cells (SW837, CMT93, A549, H1299). β-actin was used as an internal control (*n* = 3). **B** The protein levels of ACSL3, GPX4, Nrf2, SLC7A11, and TfR1 in radioresistant cancer cells were measured by Western blotting. IHC staining (**C**) and the scores (**D**) of ACSL3 protein expression in paired nonirradiated and irradiated tumor tissue samples from rectal cancer patients (*n* = 24). The upper scale bar represents 200 μm. The lower scale bar represents 100 μm. **E** The knockout efficiency of ACSL3 in SW837-RR and CMT93-RR cells was determined by western blotting. **F** CCK-8 assays were performed to determine the effects of 1 μM erastin on the viability of ACSL3-KO SW837-RR and CMT93-RR cells (*n* = 3). **G** Lipid peroxidation levels in ACSL3 KO radioresistant cancer cells. These cells were treated with DMSO (control) or 1 μM erastin (*n* = 3). **H** Quantitative analyses of GSH/GSSG ratio in ACSL3 KO radioresistant cancer cells. These cells were treated with DMSO (control) or 1 μM erastin (n = 3). *p < 0.05, **p < 0.01, ***p < 0.001.

The iron content inside and outside the cell, the lipid composition in the cell membrane and oxidative stress are strongly associated with ferroptosis. To elucidate the mechanisms underlying ferroptosis resistance in radioresistant cancer cells, we measured the mRNA expression levels of ferroptosis-associated genes in SW837-RR, CMT93-RR, A549-RR, and H1299-RR cells. ACSL3 was consistently upregulated in all four radioresistant cell lines compared with their corresponding parental cells (Fig. 4A). ACSL3 converts MUFAs into fatty acyl-CoA esters for incorporation into membrane PLs, protecting cells from ferroptosis. Furthermore, we examined the expression of several key ferroptosis-related proteins in radioresistant cancer cells, including GPX4, SLC7A11, Nrf2, TfR1, and ACSL3. Western blotting results demonstrated that ACSL3 was the only protein significantly increased in RR cancer cells (A549-RR, H1299-RR, SW837-RR, and CMT93-RR) compared with their corresponding parental cells (Fig. 4B). Importantly, ACSL3 protein levels were also significantly greater in radioresistant human primary rectal cancer tissues than in their nonirradiated controls (Fig. 4C, D). To explore the potential role of ACSL3 in irradiation-induced ferroptosis, we generated ACSL3 knockout (KO) SW837-RR and CMT93-RR cells using the CRISPR-Cas9 method (Fig. 4E). Notably, ACSL3 KO significantly enhanced the antitumor effects of erastin (or RSL3) in these cancer cells (Fig. 4F and Supplementary Fig. S3A). Accordingly, ACSL3 deficiency significantly enhanced erastin (or RSL3)-induced lipid ROS elevation, and decreased erastin-induced GSH/GSSG ratio (Fig. 4G, H and Supplementary Fig. S3B–D). Together, these results indicate that ACSL3 plays an important role in mediating ferroptosis resistance in radioresistant cancer cells.

**Fig. 5:**
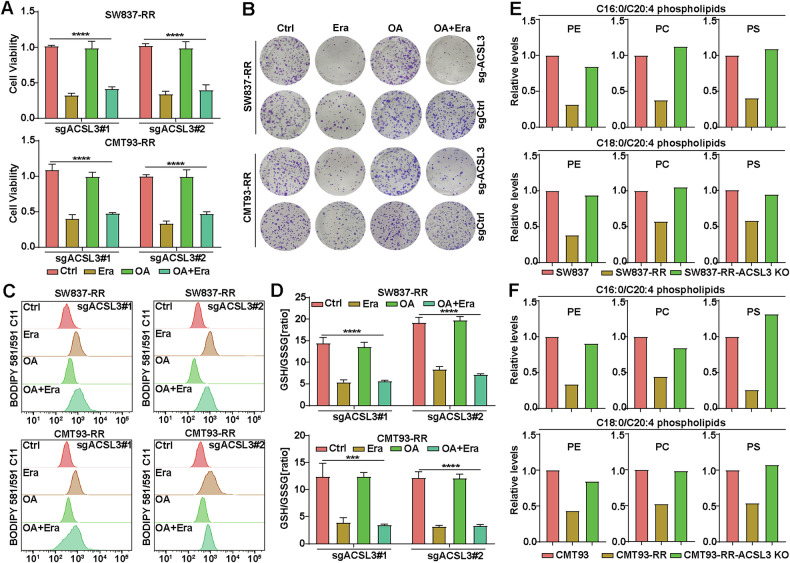
OA protects radioresistant cells from ferroptosis depending on ACSL3. **A** Viability of sgACSL3-1, sgACSL3-2 SW837-RR and CMT93-RR cells treated with or without 1 μM erastin or 20 nM OA exposure. Error bars are the means ± SD (*n* = 3). **B** Clonogenic survival assays were performed to evaluate the colony formation ability of ACSL3-KO or control cancer cells after treatment with 1 μM erastin or 20 nM OA. Student’s *t*-tests were used. Quantitative analyses of lipid peroxidation levels (**C**) and the GSH/GSSG ratio (**D**) in sgACSL3-1, sgACSL3-2 SW837-RR and CMT93-RR cells treated with or without 1 μM erastin or 20 nM OA. Lipid levels determined by mass spectrometry in SW837, SW837-RR, and SW837-RR-ACSL3 KO cells (**E**) or CMT93, CMT93-RR, and CMT93-RR-ACSL3 KO cells (**F**). PE phosphatidylethanolamine, PC phosphatidylcholine, PS phosphatidylserine. *p < 0.05, **p < 0.01, ***p < 0.001, ****p < 0.0001.

To further investigate whether OA inhibits ferroptosis through ACSL3, we evaluated the effect of OA pretreatment on the erastin (or RSL3)-induced ferroptosis in ACSL3-KO cancer cells and their parental cells. As expected, OA did not protect ACSL3-deficient cells from erastin (or RSL3) as it did in ACSL3-wild-type cells (Fig. 5A and Supplementary Fig. S4A). Next, we evaluated the impact of OA pretreatment on the clonogenic survival of a variety of erastin-treated cancer cells. The results showed that OA pretreatment blocked the inhibitory effect of erastin on the colony survival of ACSL3-deficient SW837-RR and CMT93-RR cells (Fig. 5B and Supplementary Fig. S4B). In addition, OA also failed to protect ACSL3-deficient cancer cells from erastin (or RSL3)-induced lipid ROS elevation and a decrease in the GSH/GSSG ratio (Fig. 5C, D and Supplementary Fig. S4C-E).

During ferroptosis, lipid ROS are produced by the oxidation of PUFA-PLs, such as arachidonic acid (AA) (C20:4) [12]. OA has been reported to inhibit ferroptosis by reducing the amount or density of PUFAs in cell membranes [24]. Since OA is significantly upregulated in radioresistant cancer cells and tissues, we then examined the steady-state abundance of detectable C16:0/C20:4 and C18:0/C20:4 PUFA-PLs in SW837-RR and CMT93-RR cells using mass spectrometry. Significantly decreased PUFA-PL levels were observed in these radioresistant cells, and ACSL3 deletion reversed the reduction in PUFA-PL levels (Fig. 5E, F). These data together suggest that OA protects RR cells from ferroptosis by reducing PUFA-PLs in an ACSL3-dependent manner.

### Lipid droplet (LD) formation is not required for OA to inhibit ferroptosis

**Fig. 6:**
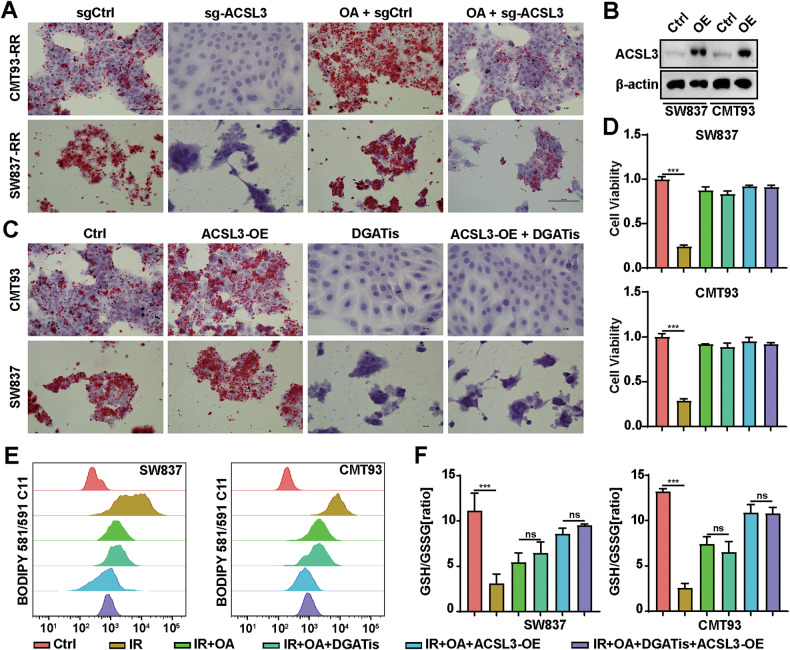
Lipid droplet (LD) formation is not required for OA to inhibit ferroptosis. **A** Oil red O staining of ACSL3 KO SW837-RR and CMT93-RR cells with or without 20 nM OA exposure. Scale bars, 50 μm. **B** The overexpression efficiency of ACSL3 in SW837 and CMT93 cells was determined by western blotting. **C** Images of Oil Red O stained ACSL3-OE SW837 and CMT93 cells exposed to DGATis (a combination of T863 (20 μM) and PF-06424439 (10 μM)). **D** CCK-8 assays were performed to determine the effects of 20 nM OA and DGATis (a combination of T863 (20 μM) and PF-06424439 (10 μM)) on the viability of ACSL3-OE SW837 and CMT93 cells. Quantitative analyses of lipid peroxidation levels (**E**) and the GSH/GSSG ratio (**F**) in OE ACSL3 SW837 and CMT93 cells treated with or without OA (20 nM) and DGATis. *p < 0.05, **p < 0.01, ***p < 0.001, ****p < 0.0001.

ACSL3 is a key enzyme responsible for free FA esterification and LD biosynthesis [28]. We evaluated whether LD formation is necessary for OA to inhibit ferroptosis. First, we determined whether ACSL3 participates in LD formation in irradiated cancer cells exposed to exogenous FAs. We found that ACSL3 KO significantly inhibited LD production, whereas OA increased LD accumulation in radioresistant cancer cells (Fig. 6A). To investigate the relationship between ACSL3-induced LD synthesis and ferroptosis, we generated stable ACSL3-overexpressing (OE) SW837 and CMT93 cells (Fig. 6B), and confirmed that ACSL3-OE significantly increased LD formation in cancer cells. Using acyl-CoA: diacylglycerol acyltransferase (DGAT) inhibitors (T863 and PF-06424439, DGATis), we specifically blocked LD formation in SW837-OE and CMT93-OE cells (Fig. 6C). However, inhibiting LD formation did not alter the OA-induced changes in cell viability, lipid ROS levels, Fe^2+^ levels or the GSH/GSSG ratio in cancer cells exposed to IR (Fig. 6D–F and Supplementary Fig. S5A, B). Thus, LD formation is not required for OA to suppress ferroptosis.

### ACSL3 knockdown in combination with IKE significantly represses radioresistance in vivo

**Fig. 7:**
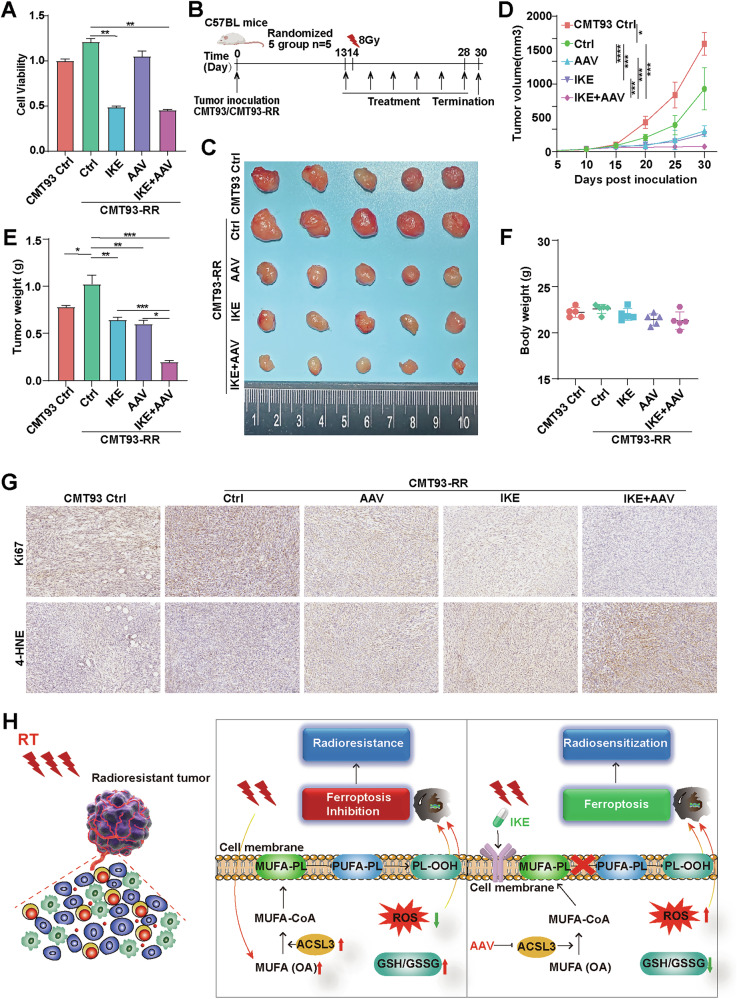
shACSL3-AAV combined with IKE significantly represses radioresistance in vivo. **A** CCK-8 assays were performed to determine the effects of 50 µM IKE and shACSL3-AAV (450,000 genome copies/cell) on the viability of CMT93-RR cells. **B** The treatment schedule for the CMT93-RR syngeneic mouse model. shACSL3-AAV was administered intratumorally at a dose of 1.8 × 10^11^ viral particles per animal. Injections were performed once every two days for a total of 6 times. Mice were treated with PBS, 30 mg/kg IKE, or shACSL3-AAV (1.8 × 10^11^ viral particles per animal) as a single agent or in combination. **C** Representative images of tumors. Tumor volume (**D**), tumor weights (**E**), and body weights of the mice (**F**). (*n* = 5) **G** The expression Ki67 and 4-HNE in the different treatment groups was measured by IHC. **H** Schematic illustration demonstrating that OA regulates ferroptosis and radioresistance in an ACSL3-dependent manner. *p < 0.05, **p < 0.01, ***p < 0.001, ****p < 0.0001.

To determine whether ferroptosis plays an important role in cancer radioresistance, we evaluated the effects of shACSL3-AAV and/or IKE on the viability of CMT93-RR cells. Due to increased potency (approximately 100-fold greater than that of erastin), solubility, and stability of the derivative of erastin IKE compared with those of erastin, IKE has been suggested to be an attractive ferroptosis inducer for in vivo preclinical studies [29]. We found that IKE obviously impaired CMT93-RR cell viability (Fig. 7A). To determine whether inducing ferroptosis could restore RT sensitivity in radioresistant cancer cells, we designed a combined therapeutic regimen of radiation, IKE and shACSL3-AAV in a CMT93-RR mouse model (Fig. 7B). As expected, both shACSL3-AAV and IKE showed a moderate growth-inhibitory effect on radioresistant tumors receiving RT. Importantly, the combination administration of shACSL3-AAV and IKE showed an obvious synergistic anticancer effect, which enhanced the sensitivity of radioresistant tumors to RT more significantly than the other treatments (Fig. 7C–F). Moreover, significantly decreased expression of Ki67 and increased expression of 4-HNE (a lipid peroxidation marker) were observed in the IKE+shACSL3-AAV compared with the other groups, further supporting that this synergistic effect promoted the induction of tumor ferroptosis (Fig. 7G). Taken together, these results demonstrate that combining IKE and shACSL3-AAV provides a novel strategy to overcome cancer radioresistance.

## Discussion

It is challenging to improve the prognosis of advanced patients with refractory tumors resulting from radioresistance [2]. Considering that lipid metabolism in cancer cells, and ferroptosis are closely related to radiation, uncovering new mechanisms regulating these processes will lead to the identification of novel therapeutic targets to overcome radioresistance. In this study, for the first time, we found that the biosynthesis of MUFAs, especially OA, is increased in radioresistant cancer cells. In addition, ACSL3, an enzyme catalyzing esterification of MUFAs with coenzyme A, is also upregulated in radioresistant cancer cells. Mechanistically, OA promotes ferroptosis resistance in radioresistant cancer cells in an ACSL3-dependent manner via a reduction in the amount of PUFA-PLs in cell membranes and inhibition of lipid peroxidation and subsequent ferroptosis, leading to cancer radioresistance. IKE combined with shACSL3-AAV, had significant synergistic antitumor effects and reversed radioresistance in radioresistant cancer cells (Fig. 7H).

In recent years, the contribution of ferroptosis to the efficacy of RT or radiosensitization has attracted increased amounts of attention [3, 8, 9]. For example, IR induces ferroptosis partly through the upregulation of ACSL4 [10]. IR also depletes intracellular GSH, which impairs the antiferroptotic effect mediated by GPX4 and further promotes ferroptosis [30]. Inactivation of SLC7A11 or GPX4 can restore the radiosensitivity of radioresistant cancer cells and xenograft tumors [10, 31]. In addition, immune cells, especially CD8^+^ T cells, may participate in RT-induced ferroptosis. IFN-γ produced by immunotherapy-activated CD8^+^ T cells promotes tumor ferroptosis and induces radiosensitization. RT-activated ATM and IFN-γ-induced STAT1 signaling jointly repress SLC7A11 to reduce cystine uptake and enhance lipid peroxidation and ferroptosis in tumors [11]. However, the detailed mechanisms linking ferroptosis and radioresistance are still unclear.

In the last decade, several FA metabolic pathways have been suggested to regulate ferroptosis sensitivity [32, 33]. Unfortunately, cancer cells that are initially sensitive to ferroptosis can also switch to a ferroptosis-resistant state [34–37]. Thus, the following question arises: which FA metabolic pathways are involved in regulating ferroptosis sensitivity and radiosensitivity? Recent studies have reported that IR can change the FA profile in breast cancer [38]. Martino et al [22] reported that RT induces lipogenic enzymes to accumulate the majority of unsaturated FAs, such as OA (C18:1), docosahexaenoic acid (DHA; C22:6), and arachidonic acid (C20:4), in glioblastoma cells. Notably, blocking fatty acid synthase (FASN) obviously promoted the efficacy of focal RT in tumor-bearing mice [22]. Other studies also showed that inhibiting FASN could sensitize cancer cells to RT [39, 40]. These studies suggested that FA metabolism may regulate radioresistance via ferroptosis. In this study, we found that OA and POA levels were increased in radioresistant tumor cells and played a crucial role in mediating the radioresistance in these cells, which is consistent with previous observations that abundant OA protects tumor cells from ferroptosis and promotes metastasis in melanoma [27].

The ACSL family contains five isoforms (ACSL1, 3, 4, 5, and 6) that convert free long-chain FAs into fatty acyl-CoA esters, playing key roles in both the anabolic (FA synthesis and lipogenesis) and catabolic (lipolysis and FA β-oxidation) pathways [41]. Among these five isoforms, ACSL3 and ACSL4 have been shown to participate in ferroptosis [42]. For example, ACSL4 is a positive regulator of ferroptosis, whereas ACSL3 contributes to the acquisition of ferroptosis resistance in cancer cells. However, little is known about the relationship between IR and the ACSL family. Our data revealed that ACSL3 was obviously increased in radioresistant cancer cells. ACSL3 can catalyze the conversion of MUFAs to MUFA-CoA to increase MUFA-PLs and decrease PUFA-PLs in cell membranes, inhibiting lipid peroxidation and subsequent ferroptosis. Excess FAs induced by IR lead to lipotoxicity, which causes cell death, and irradiated cancer cells can balance FA metabolism by producing LD to counteract lipotoxicity and protect cancer cells from RT [22]. In addition, ACSL3 is also a key factor contributing to the production of LD. However, although we also observed increased LD formation in OA-treated cancer cells and in ACSL3-OE cancer cells, LD formation is not necessary for OA/ACSL3 to suppress ferroptosis, highlighting that their regulation on PUFA-PLs is the key to inducing ferroptosis and radioresistance in cancer cells. Importantly, we demonstrated that the combination of shACSL3-AAV with IKE has excellent synergistic antitumor effects in radioresistant tumor cells response to RT, suggesting that targeting ACSL3 and inducing ferroptosis cooperate to resensitize radioresistant cancer cells to radiation, suggesting a promising prospect for future clinical translation.

There are several limitations to this study. First, the suppressive effect of ACSL3 on ferroptosis needs to be further studied, particularly the interaction between OA and ACSL3. In addition, the role of ACSL3 in the biological effect of high-dose and low-dose irradiation should be elucidated in further studies.

We revealed for the first time the relationships between radioresistance and FAs, ACSL3, and ferroptosis, and elucidated the underlying mechanism through which MUFAs modulate radioresistance in tumor cells. The present study demonstrated that IKE can reverse the radioresistance of tumor cells. Notably, we also found that silencing ACSL3 expression, exhibited therapeutic efficacy in combination with IKE in vivo, laying a solid foundation for future clinical applications. Consequently, we propose that targeting ACSL3 and inducing ferroptosis should be considered a novel therapeutic strategy for overcoming cancer radioresistance.

## Supplementary information

Supplementary information

western blots

## Data Availability

The data that support the findings of this study are available upon reasonable request. The original full size western blots are shown in the Original Data WB File.
