# Correction: Monounsaturated fatty acids promote cancer radioresistance by inhibiting ferroptosis through ACSL3

**DOI:** 10.1038/s41419-025-07726-6

**Published:** 2025-06-03

**Authors:** Yulin Cao, Jiuming Li, Ying Chen, Yuanben Wang, Zhiang Liu, Liuying Huang, Bingxin Liu, Yuyang Feng, Surui Yao, Leyuan Zhou, Yuan Yin, Zhaohui Huang

**Affiliations:** 1 Wuxi Cancer Institute, Affiliated Hospital of Jiangnan University, Wuxi, Jiangsu 214062 China; 2 Laboratory of Cancer Epigenetics, Wuxi School of Medicine, Jiangnan University, Wuxi, Jiangsu China; 3 Department of Radiation Oncology, Affiliated Hospital of Jiangnan University, Wuxi, Jiangsu China

**Keywords:** Cancer therapeutic resistance, Radiotherapy, Rectal cancer, Oncogenes

Correction to: *Cell Death & Disease* 10.1038/s41419-025-07516-0, published online 18 March 2025

The original version of this article contained an error in Figure 5B. The authors state the following: “The representative clone image for the OA+Era of the SW837-RR-sgCtrl group in Fig. 5B was misused due to an inadvertent layer superimposition during the figure assembly”. This image in Figure 5B was replaced with a correct one. The corrected image can be found below.

**Original Figure 5B** 
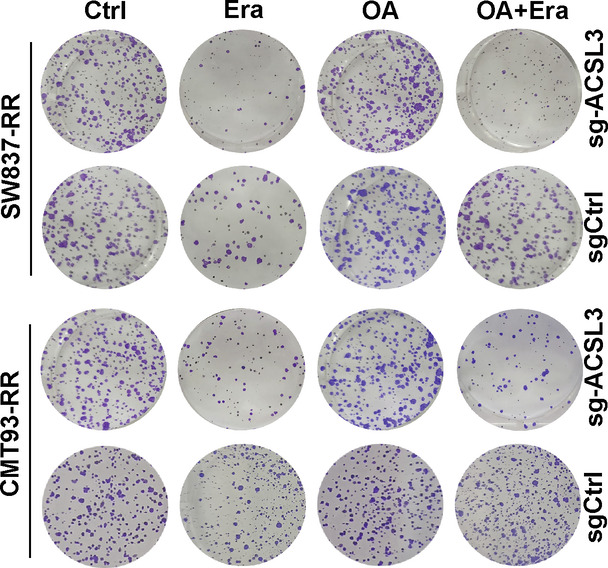


**Amended Figure 5B** 
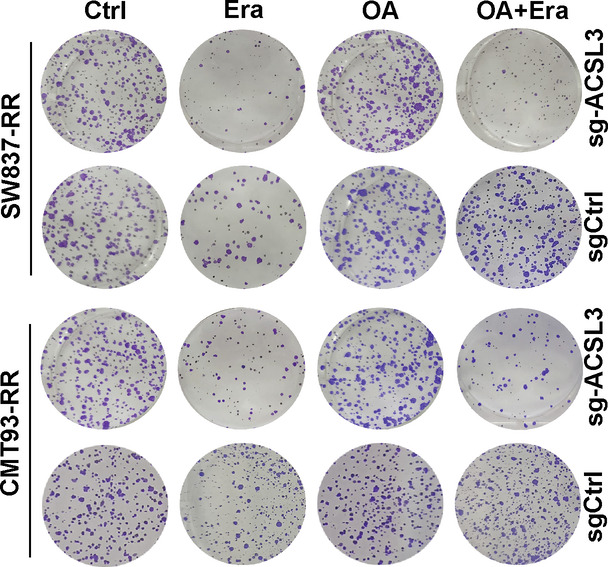


The correction does not affect the conclusions of the above paper. The authors apologize for the mistake and any inconvenience caused.

The original article has been corrected.

