# Supplementary material for: Monounsaturated fatty acids promote cancer radioresistance by inhibiting ferroptosis through ACSL3
Source: Cell Death Dis. 2025 Mar 18;16(1):184. doi: 10.1038/s41419-025-07516-0 (PMC11920413; doi:10.1038/s41419-025-07516-0)
Supplement: Supplementary file 1 — Supplementary information [file 41419_2025_7516_MOESM1_ESM.docx]

**Supplementary information**

**Monounsaturated fatty acids promote cancer radioresistance by inhibiting ferroptosis through ACSL3**

Yulin Cao^1,2^, Jiuming Li^1,2^, Ying Chen^1,2^, Yuanben Wang^1,2^ , Zhiang Liu^1,2^, Liuying Huang^1,2^, Bingxin Liu^1,2^, Yuyang Feng^1^, Surui Yao^1,2^, Leyuan Zhou^2,3^, Yuan Yin^1,2,*^, Zhaohui Huang^1,2,*^

**Supplementary Figure 1-5:**

**
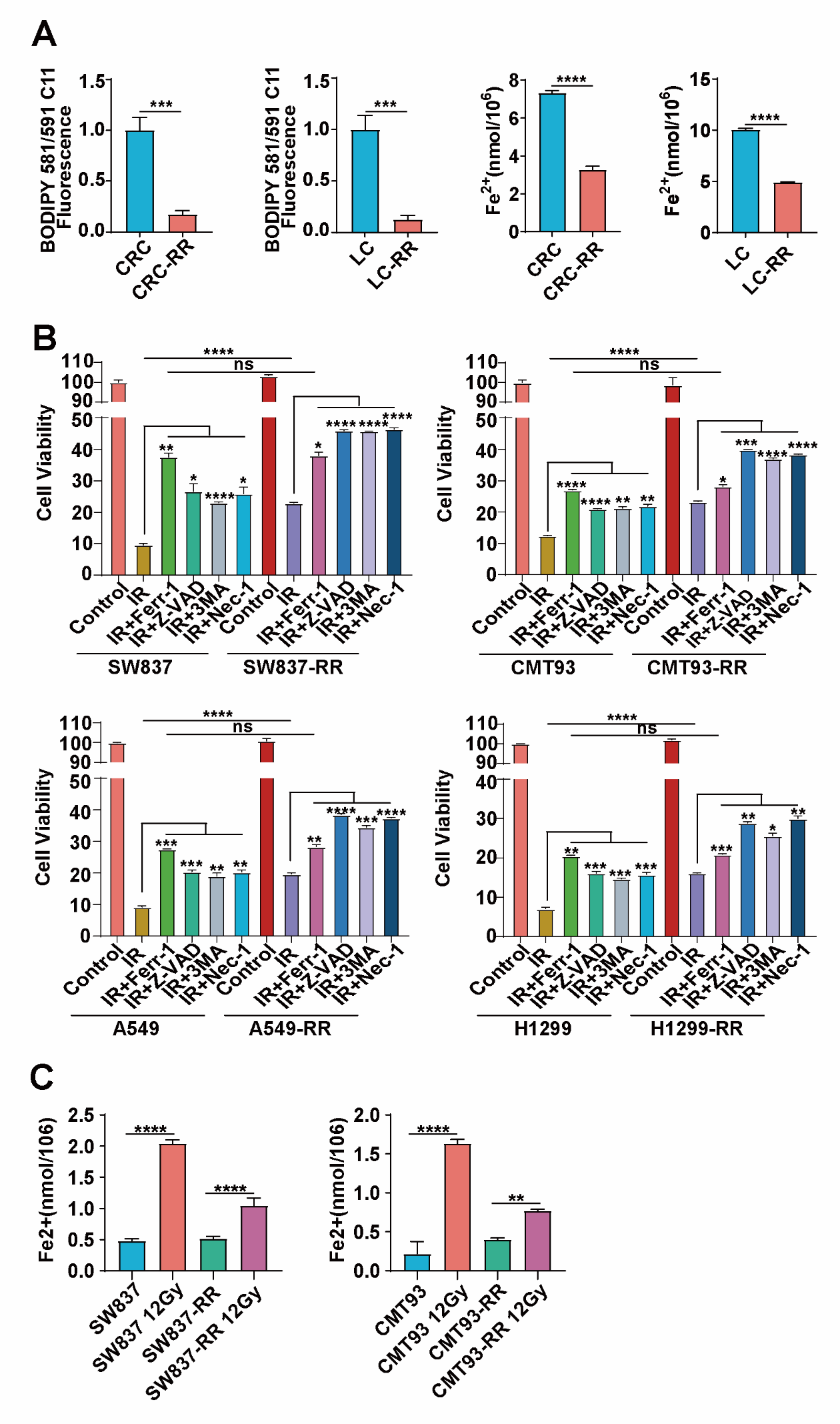
**

**Figure S1 A** Quantitative analyses of lipid ROS and Fe^2+^ level in nonirradiated control tumors and radioresistant tissues (RR) after RT. **B** Cell viability assays in cancer cells and their radioresistant cells pretreated with 5 μM ferrostatin-1, 5 μM Z-VAD-fmk, 2 μM necrostatin-1, and 5 mM 3-methyladenine (3-MA), or DMSO for 24 h followed by exposure to IR. The cell survival data were normalized to those of unirradiated control cells. Error bars are the means ± SD, n = 3 independent repeats. **C** Quantitative analyses of Fe^2+^ levels in reirradiated radioresistant cancer cells and control cells. **p* < 0.05, ***p* < 0.01, ****p* < 0.001, *****p* < 0.0001.


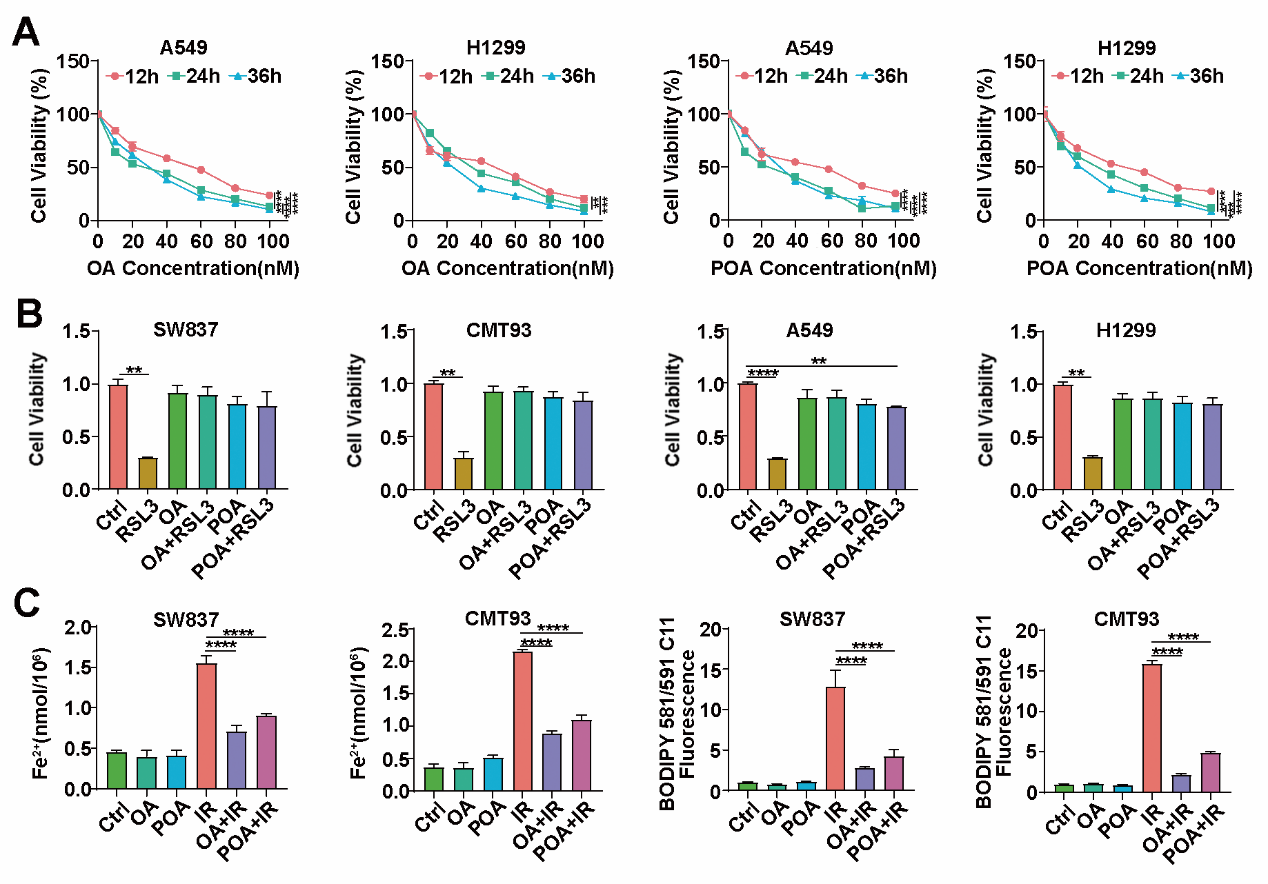


**Figure S2 A** The cell cytotoxicity of different concentrations (0-100 nM) of OA and POA against A549 and H1299 cells at 12 h, 24 h, and 36 h. **B** CCK-8 assays were performed to determine the effects of RSL3 (0.5 μM), OA (20 nM), and POA (20 nM) on the viability of cancer cells (n=3). **C** Quantitative analyses of lipid ROS and Fe^2+^ level in cancer cells treated with IR, 20 nM OA, or 20 nM POA. **p* < 0.05, ***p* < 0.01, ****p* < 0.001, *****p* < 0.0001.


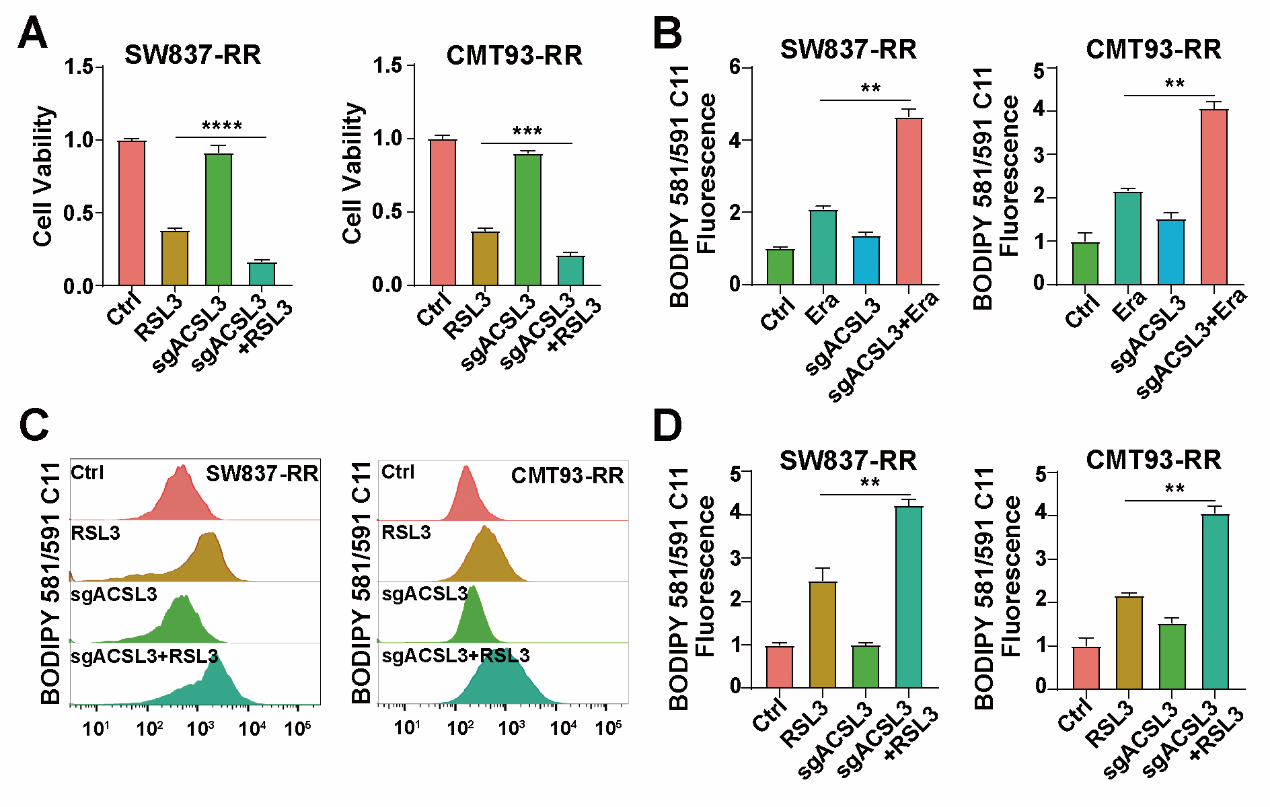


**Figure S3** **A** CCK-8 assays were performed to determine the effects of 0.5 μM RSL3 on the viability of ACSL3-KO SW837-RR and CMT93-RR cells (n=3). **B** Quantitative analyses of lipid ROS in radioresistant cancer cells with ACSL3 KO. These cells were treated with DMSO (control) or 1 μM erastin. **C-D** Quantitative analyses of lipid ROS in radioresistant cancer cells with ACSL3 KO. These cells were treated with DMSO (control) or 0.5 μM RSL3. **p* < 0.05, ***p* < 0.01, ****p* < 0.001, *****p* < 0.0001.

**
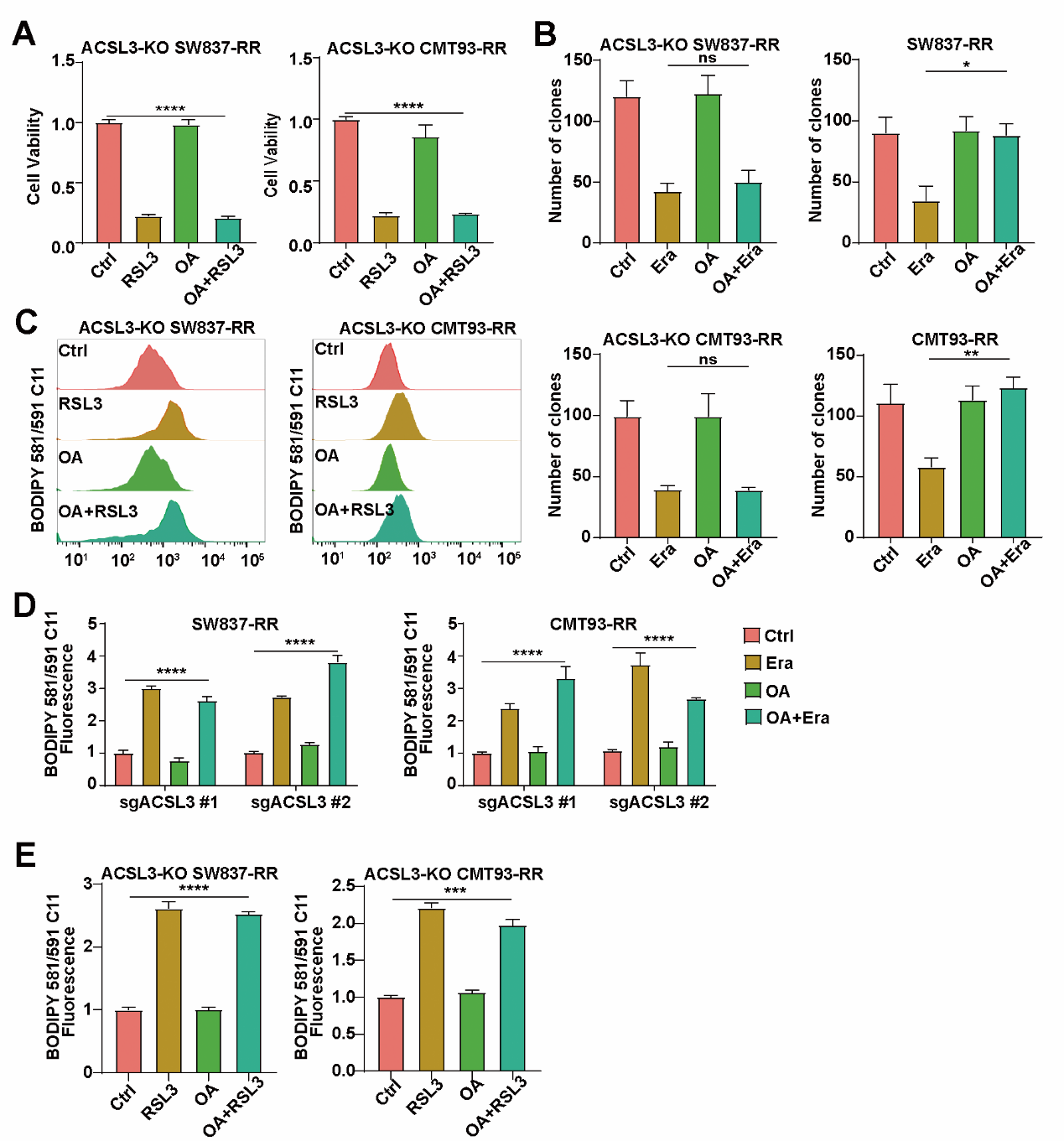
**

**Figure S4** **A** Viability of sgACSL3-1, sgACSL3-2 SW837-RR and CMT93-RR cells treated with 0.5 μM RSL3 or 20 nM OA exposure. Error bars are the means ± SD. **B** Clonogenic survival assays were performed to evaluate the colony formation ability of ACSL3-KO or control cells after exposure to 1 μM erastin or 20 nM OA. The data are representative of three independent experiments and are presented as the means ± SD; Student’s *t*-tests were used. **C** Lipid peroxidation levels in ACSL3 KO radioresistant cancer cells. These cells were treated with DMSO (control) or 0.5 μM RSL3 (n=3). **D** Quantitative analyses of lipid ROS in sgACSL3-1, sgACSL3-2 SW837-RR and CMT93-RR cells treated with 1 μM erastin or 20 nM OA. **E** Quantitative analyses of lipid ROS in ACSL3-KO SW837-RR and CMT93-RR cells treated with or without 0.5 μM RSL3 or 20 nM OA.**p* < 0.05, ***p* < 0.01, ****p* < 0.001, *****p* < 0.0001.

**
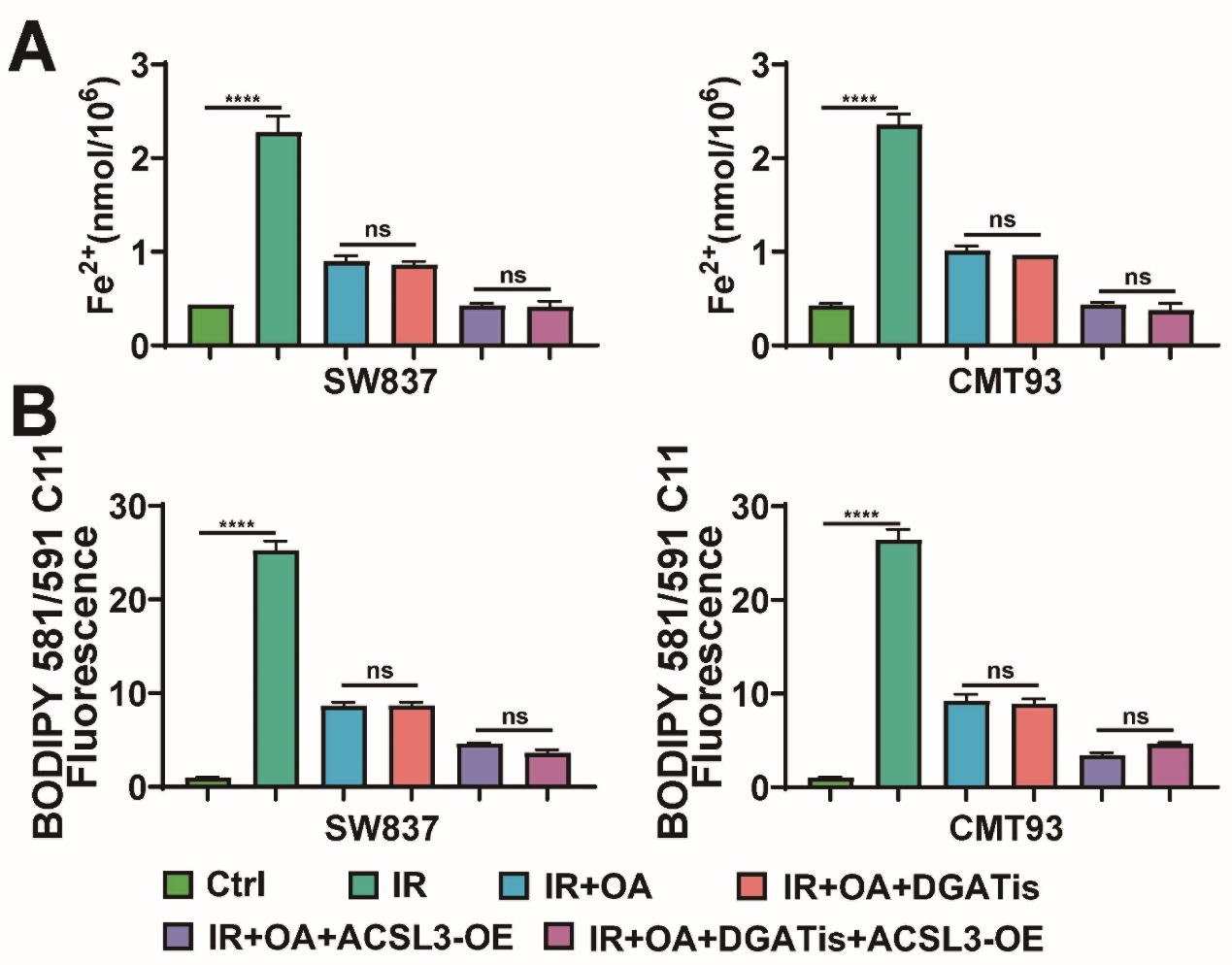
**

**Figure S5 A-B** Quantitative analyses of Fe^2+^ level (**A**) and lipid ROS (**B**) in OE ACSL3 SW837 and CMT93 cells treated with or without OA (20 nM) and DGATis. **p* < 0.05, ***p* < 0.01, ****p* < 0.001, *****p* < 0.0001.

**Supplementary Table 1:** List of primers.

| Primers for CRISPR-Cas9 assay | | | | | |
| --- | --- | --- | --- | --- | --- |
| Gene | Forward Primer (5’>3’ ) | | Reverse Primer (5’>3’) | | |
| ACSL3sgRNA#H1 | CACCG AGCTATCATCCACTCGGCCC | | AAAC GGGCCGAGTGGATGATAGCTC | | |
| ACSL3sgRNA#H2 | CACCG CGAGTGGATGATAGCTGCAC | | AAAC GTGCAGCTATCATCCACTCGC | | |
| ACSL3sgRNA#M1 | CACCG GAGTCCGGTTTGGAACTGAC | | AAAC GTCAGTTCCAAACCGGACTCC | | |
| ACSL3sgRNA#M2 | CACCG TGAGTGGATGATCGCTGCAC | | AAAC GTGCAGCGATCATCCACTCAC | | |
| primers for qRT-PCR | | | | |  |
| Gene | | Forward Primer (5’>3’ ) | | Reverse Primer (5’>3’) |  |
| ACSL3 | | CTGCAGTTGTCTACGCGGC | | CGAATTCTGTATCGCTACGCC |  |
| ACTB | | AGTGTGACGTGGACATCCGCAAAG | | ATCCACATCTGCTGGAAGGTGGAC |  |
| GPX4 | | CGATACGCTGAGTGTGGTTTGC | | CATTTCCCAGGATGCCCTTG |  |
| ACSL4 | | CATCCCTGGAGCAGATACTCT | | TCACTTAGGATTTCCCTGGTCC |  |
| FSP1 | | ACATGGTGAGGCAGGTCCA | | GCCACTTGGGAGTGAATGAG |  |
| SLC7A11 | | TTGTTTTGCACCCTTTGACA | | AAAGCTGGGATGAACAGTGG |  |
