# Supplementary material for: Monounsaturated fatty acids promote cancer radioresistance by inhibiting ferroptosis through ACSL3
Source: Cell Death Dis. 2025 Mar 18;16(1):184. doi: 10.1038/s41419-025-07516-0 (PMC11920413; doi:10.1038/s41419-025-07516-0)
Supplement: Supplementary file 2 — western blots [file 41419_2025_7516_MOESM2_ESM.pptx]

## Slide 1
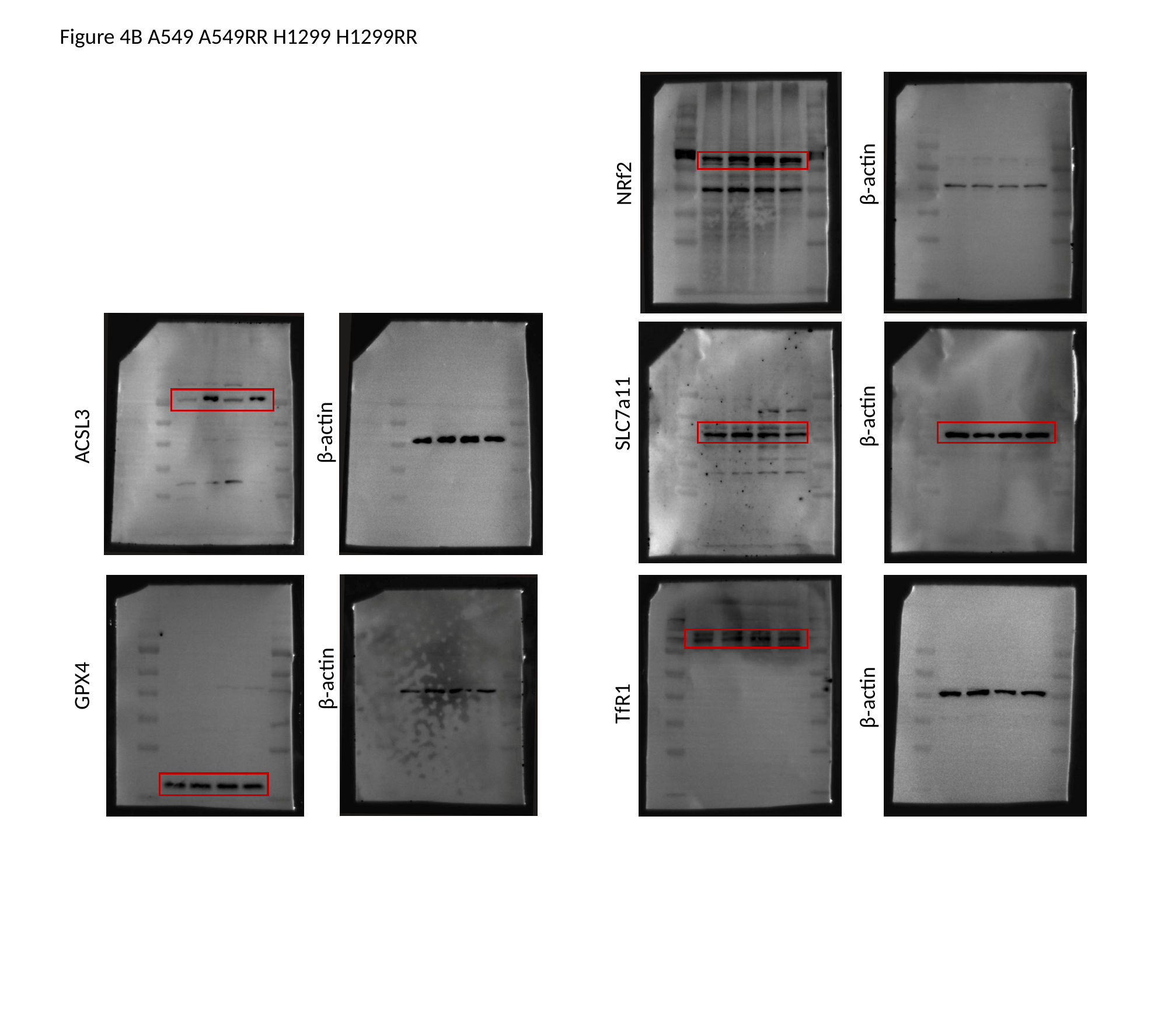

Figure 4B A549 A549RR H1299 H1299RR
NRf2
β-actin
β-actin
SLC7a11
ACSL3
β-actin
GPX4
β-actin
TfR1
β-actin

## Slide 2
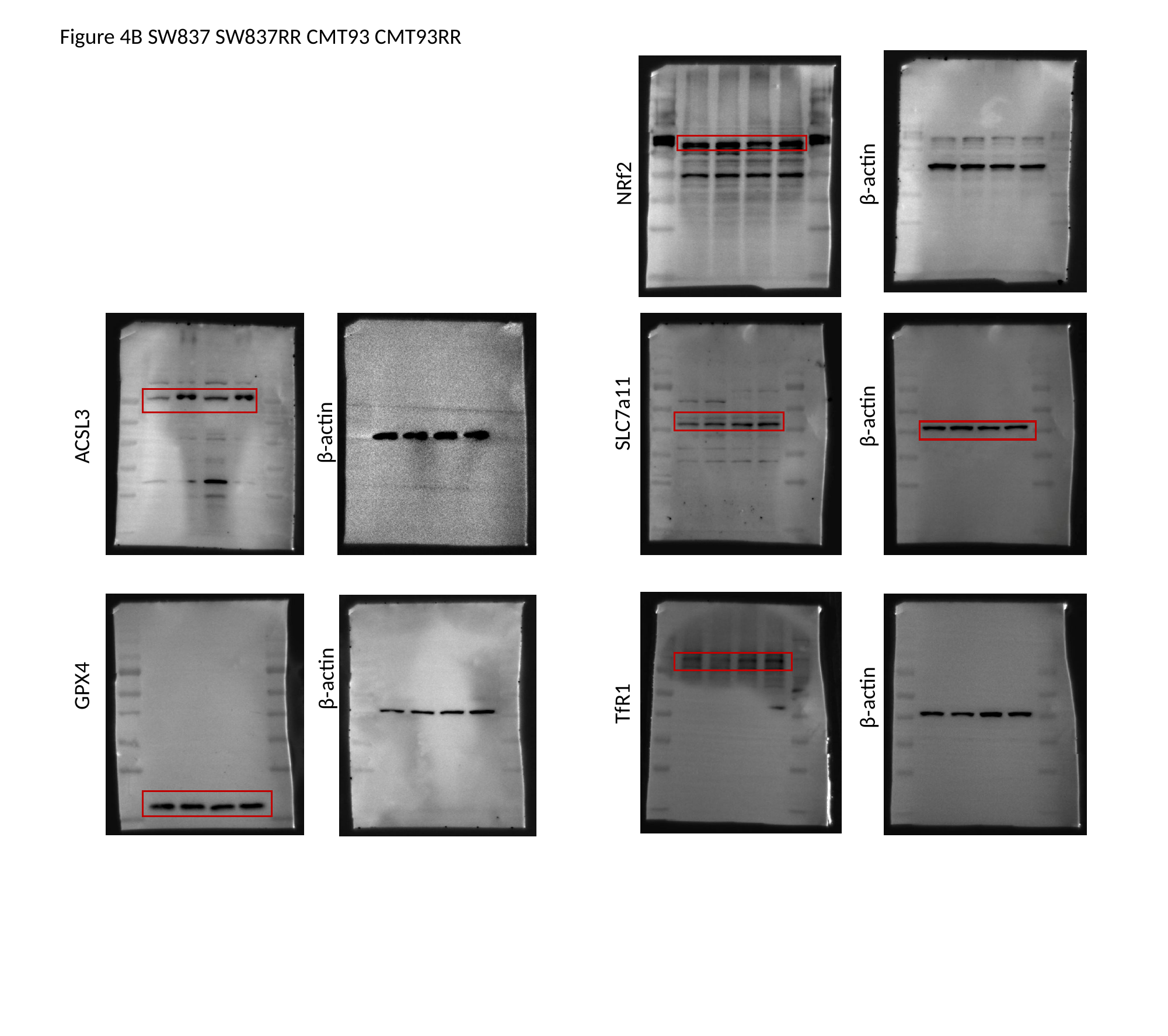

Figure 4B SW837 SW837RR CMT93 CMT93RR
NRf2
β-actin
β-actin
SLC7a11
ACSL3
β-actin
GPX4
β-actin
TfR1
β-actin

## Slide 3
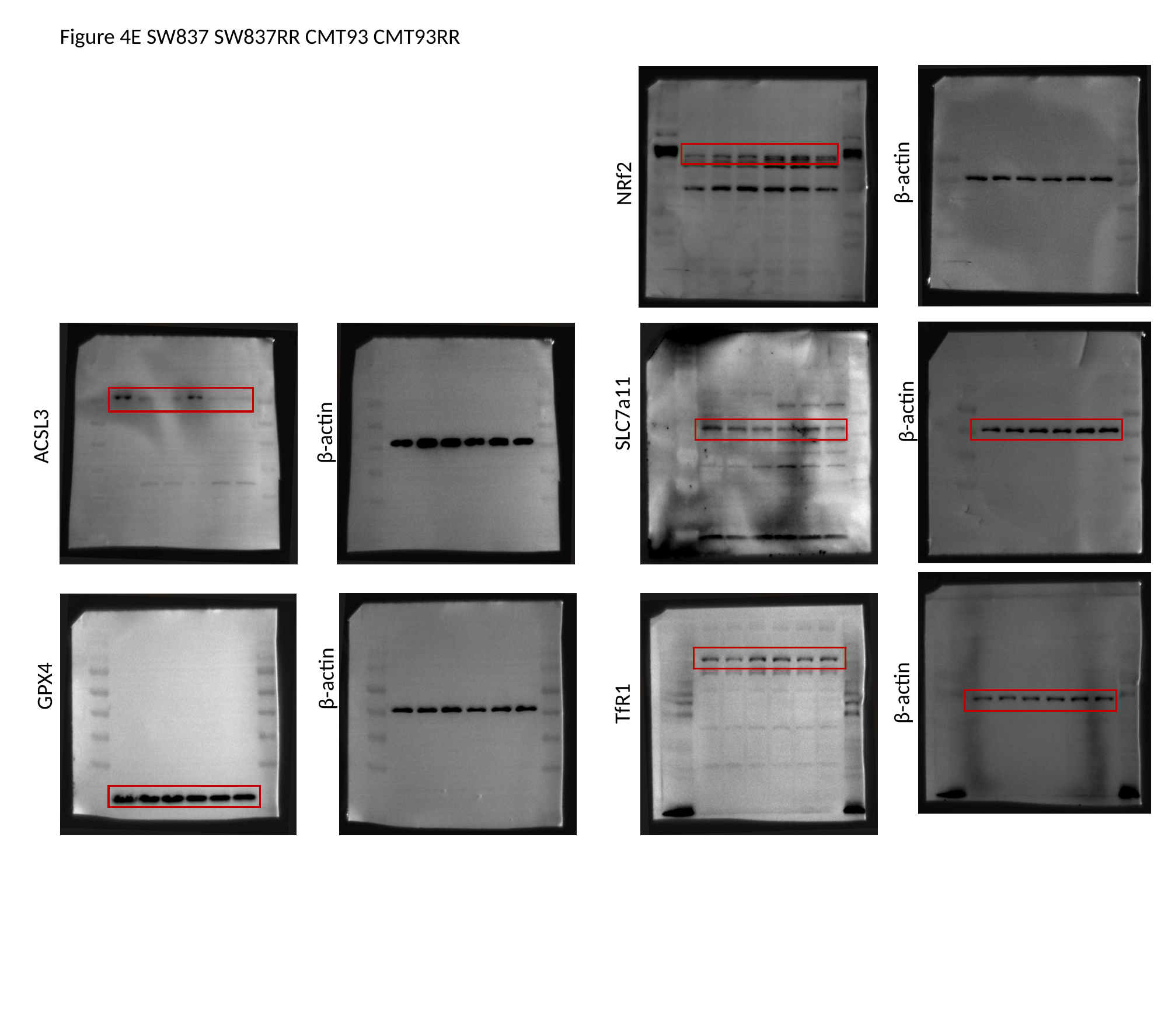

Figure 4E SW837 SW837RR CMT93 CMT93RR
β-actin
NRf2
β-actin
SLC7a11
ACSL3
β-actin
GPX4
β-actin
TfR1
β-actin

## Slide 4
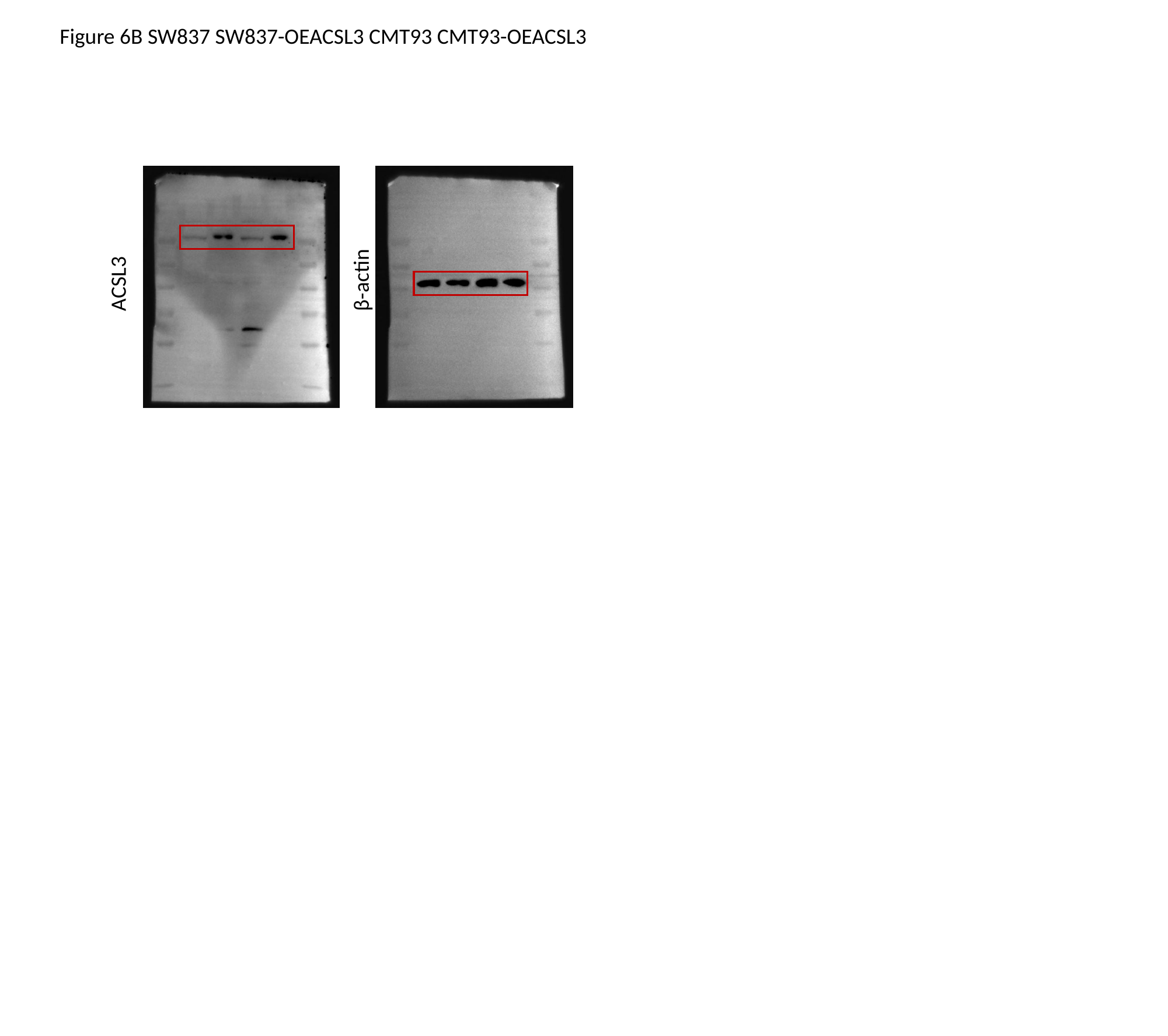

Figure 6B SW837 SW837-OEACSL3 CMT93 CMT93-OEACSL3
ACSL3
β-actin
